# Construction and validation of a risk prediction model for extrauterine growth restriction in preterm infants born at gestational age less than 34 weeks

**DOI:** 10.3389/fped.2024.1381193

**Published:** 2024-09-18

**Authors:** Yu Xie, Zhihui Zhang, Mengmeng Luo, Yan Mo, Qiufen Wei, Laishuan Wang, Rong Zhang, Hanlu Zhong, Yan Li

**Affiliations:** ^1^ Ruikang Clinical Medical College, Guangxi University of Chinese Medicine, Nanning, China; ^2^ JC School of Public Health and Primary Care, The Chinese University of Hong Kong, Shatin, NT, Hong Kong SAR, China; ^3^ Department of Applied Mathematics, The Hong Kong Polytechnic University, Kowloon, Hong Kong SAR, China; ^4^ Department of Biological Sciences, University of Liverpool, Liverpool, United Kingdom; ^5^ Neonatal Medical Centre, Maternity and Child Health Hospital of Guangxi Zhuang Autonomous Region, Nanning, China; ^6^ Guangxi Clinical Research Center for Pediatric Diseases, Nanning, China; ^7^ Department of Neonatology, Children’s Hospital of Fudan University, National Children’s Medical Center, Shanghai, China

**Keywords:** preterm infants, EUGR, risk prediction model, random forest, LASSO regression

## Abstract

**Objective:**

This study aimed to develop and validate a model for predicting extrauterine growth restriction (EUGR) in preterm infants born ≤34 weeks gestation.

**Methods:**

Preterm infants from Guangxi Maternal and Child Health Hospital (2019–2021) were randomly divided into training (80%) and testing (20%) sets. Collinear clinical variables were excluded using Pearson correlation coefficients. Predictive factors were identified using Lasso regression. Random forest (RF), support vector machine (SVM), and logistic regression (LR) models were then built and evaluated using the confusion matrix, area under the curve (AUC), and the F1 score. Additionally, calibration curves and decision curve analysis (DCA) were plotted to assess the performance and practical utility of the models.

**Results:**

The study included 387 infants, with no significant baseline differences between training (*n *= 310) and testing (*n *= 77) sets. LR identified gestational age, birth weight, premature rupture of membranes, patent ductus arteriosus, cholestasis, and neonatal sepsis as key EUGR predictors. The RF model (19 variables) demonstrated an accuracy of greater than 90% during training, and superior AUC (0.62), F1 score (0.80), and accuracy (0.72) in testing compared to other models.

**Conclusions:**

Gestational age, birth weight, premature rupture of membranes, patent ductus arteriosus, cholestasis, and neonatal sepsis are significant EUGR predictors in preterm infants ≤34 weeks. The model shows promise for early EUGR prediction in clinical practice, potentially enhancing screening efficiency and accuracy, thus saving medical resources.

## 1 Introduction

According to statistics from 2016, approximately 16% of global mortality and 35% of neonatal deaths are attributable to preterm birth (1). Research conducted by the WHO indicates that preterm birth is a leading cause of mortality among children under five globally (2). Advancements in medical care and health conditions, particularly in medical technology within Neonatal Intensive Care Units (NICUs), have positively impacted the survival rates of preterm infants (3). However, surviving preterm infants continue to face a range of short-term and long-term health challenges (4), including developmental issues related to growth restriction or delay, such as bronchopulmonary dysplasia, necrotizing enterocolitis, and feeding difficulties (5). These challenges are exacerbated by perinatal conditions, the NICU environment, and nutritional support.

The concept of Extrauterine Growth Restriction (EUGR) was first proposed by Clark et al. in 2003 (6). They observed that during hospitalization, preterm infants were significantly affected by EUGR in terms of weight (28%), length (34%), and head circumference (16%) (6). Multiple studies indicate that EUGR affects 40% to 95% of premature infants, increasing their susceptibility to various diseases and contributing to adverse prognostic outcomes (7). EUGR is associated with alterations in cardiac metabolism and inflammatory states (8), correlating with poor metabolic and neurodevelopmental outcomes (9). Some severely affected premature infants with EUGR exhibit growth retardation during childhood (10).

The etiology of EUGR is complex, involving interactions among various factors such as pregnancy and environmental influences (11), as well as the potential for NICU exposure to predispose premature infants to multiple diseases (12, 13). Radmacher, Paula G. et al. developed a logistic regression model to predict the occurrence of EUGR in extremely low birth weight infants (14). However, their model included a limited number of predictive variables, excluding potential genetic and environmental factors, and relied solely on traditional statistical methods (14).

In the current context of increasing popularity of machine learning in healthcare, risk prediction models offer a rapid and effective means to assess individual disease susceptibility (15–17). These models estimate the probability of an individual developing a particular condition or experiencing specific outcomes based on a range of individual characteristics. They are commonly used in clinical settings to stratify disease severity and identify risk factors for disease or prognosis. The rapid, convenient, and effective assessments provided by these models are crucial for initial clinical analysis and diagnosis. Compared to traditional statistical methods, machine learning algorithms do not require data to adhere to specific statistical assumptions, such as independence of observations and avoidance of multicollinearity (18, 19).

In this study, predictive variables for early onset of EUGR in premature infants were selected, and three predictive were constructed: Random Forest (RF), Logistic Regression (LR), and Support Vector Machine (SVM). By comparing the performance and accuracy of these models, the optimal predictive model suitable for this study was identified. The aim is to facilitate early identification of EUGR, prompt intervention, and improvement in prognosis.

## 2 Methods

### 2.1 Study design and population

This study was approved by the Ethics Committee of the Maternal and Child Health Hospital of Guangxi Zhuang Autonomous Region [(2019-4) NO.4]. A total of 387 preterm infants admitted to the NICU of the hospital from January 1, 2019, to December 31, 2021, and subsequently discharged based on medical advice, were selected as study subjects. The dataset was randomly divided into training and testing sets in an 8:2 ratio. In the training set, clinical variables with collinearity were excluded based on Pearson correlation coefficients. Lasso regression and logistic regression (LR) were used to analyze and select predictive factors. Subsequently RF, SVM, and MLR were employed to construct risk prediction models for EUGR in preterm infants. The performance of these prediction models was comprehensively evaluated using the confusion matrix, receiver operating characteristic (ROC) curve, and F1 score in the testing set, and the optimal prediction model was ultimately selected.

Inclusion criteria: (1) admission to our NICU; (2) gestational age ≤34 weeks; (3) postnatal age at admission <24 h; (4) hospital stay ≥7 days; (5) stable vital signs and no need for respiratory support at discharge; (6) able to tolerate full oral feeding at discharge; (7) informed consent obtained from the family.

Exclusion criteria: (1) multiple births; (2) congenital malformations requiring surgical treatment that affects nutrient intake, such as gastrointestinal abnormalities or tracheoesophageal fistula; (3) congenital heart disease, chromosomal disorders, or genetic metabolic diseases; (4) died during hospitalization; (5) discharged against medical advice; (6) incomplete clinical data.

Definitions: EUGR refers to growth parameters (weight, height, head circumference) below the 10th percentile for the same gestational age at discharge (6). Birth weight is defined as the weight of the neonate recorded within 1 hour after birth (20). Neonatal sepsis is defined as either bacterial or fungal sepsis (21, 22). Neonatal respiratory distress syndrome is diagnosed according to the 2022 European guidelines (23). Retinopathy of prematurity (ROP) is defined as a retinal disease characterized by abnormal proliferation of retinal vessels (24). Necrotizing enterocolitis (NEC) is defined according to Bell's criteria (25). The diagnosis and grading of intraventricular hemorrhage are based on Papile's criteria (26). Antenatal corticosteroid use is defined as the administration of corticosteroids to the pregnant mother at least 48 hours before delivery (27). The definitions of gestational hypertension syndrome, gestational diabetes mellitus, and premature rupture of membranes are based on obstetrics literature (28). Clinical diagnosis of chorioamnionitis is established if any one of the following criteria is met: maternal fever (temperature >38°C) with maternal tachycardia (heart rate >100 bpm), fetal tachycardia (fetal heart rate >160 bpm), uterine tenderness, foul-smelling amniotic fluid, or elevated maternal white blood cell (WBC) count (29).

### 2.2 Data collection

Data were systematically collected from the inpatient medical records, including general information (gender, gestational age, birth weight, mode of delivery, etc.), parental characteristics (maternal age, parental education level, method of conception, etc.), relevant risk factors of maternal infections during pregnancy (premature rupture of membranes, chorioamnionitis, antenatal steroid use, etc.), and postnatal conditions of preterm infants (hyperbilirubinemia, neonatal sepsis, bronchopulmonary dysplasia, etc.). Clinical data collection was independently performed by two individuals and subsequently verified to ensure quality control.

### 2.3 Constructing and comparing predictive models

#### 2.3.1 Screening predictive factors

In the training set, potential collinearity among the variables was assessed by calculating Pearson correlation coefficients (r ≥ 0.7 indicating strong correlation). Variables exhibiting significant correlations were considered for exclusion based on a literature review and consultations with neonatology experts. To address multicollinearity in clinical variables, the Lasso regression method was employed. This method is effective for selecting variables with higher interpretability, managing high-dimensional data, and mitigating multicollinearity, ensuring the model's reliability and generalizability. Variables were selected using penalty coefficients (*λ*), which apply penalties to predictive factors. A larger *λ* imposes stricter selection criteria, resulting in fewer predictive factors in the final model. Setting *λ* to one standard error, the model effectively performs with fewer predictive factors. Additionally, LR analysis was conducted to validate the predictive factors by identifying significant variables.

#### 2.3.2 Construct risk predictive models

Using the training set and selected predictive variables, we constructed three models to predict EUGR in preterm infants born at ≤34 weeks gestational age. We employed the bootstrap resampling method, conducting 380 iterations on the training set to generate multiple bootstrap samples. Each bootstrap sample was used to train 500 individual decision trees, resulting in the formation of a RF prediction model (Supplementary Figure S2). Analyzing the Gini coefficient helped identify the most important features, simplifying the model and enhancing its predictive performance. Additionally, based on the training set, we used the LR algorithm to train the predictive model, while determining the optimal decision boundary for the SVM model.

#### 2.3.3 Evaluate model performance

Model performance was evaluated using the area under the ROC curve (AUC) to assess discrimination (30). Sensitivity, specificity, and F1 score were computed for the three models in the testing set. Confusion matrices were used to determine the accuracy of the random forest model on both the training and testing sets, aiding in the evaluation of the model's generalization ability and predictive accuracy. The optimal predictive model was selected based on a comprehensive assessment of multiple performance metrics. Calibration curves were generated using the training set to evaluate the model's probability prediction accuracy (31). Additionally, decision curve analysis (DCA) was performed to assess the model's practical utility in clinical settings (32).

### 2.4 Statistical analysis

Statistical analysis and model construction were performed using R (version 4.1.1). Categorical variables were expressed as percentages, while continuous variables were described using means and standard deviations (SD) to represent central tendency and variability. A *p*-value of less than 0.05 was considered statistically significant for two-tailed tests. Confusion matrices were generated, and metrics such as the F1 score and AUC were calculated to analyze and evaluate the predictive models.

## 3 Results

### 3.1 General characteristics

A total of 526 eligible preterm infants were enrolled in this study. Of these, 16 infants passed away during hospitalization, and 55 were discharged against medical advice. Among the surviving infants, 68 were lost to follow-up. Consequently, the study included a total of 387 preterm infants (Supplementary Figure S1), with 310 in the training set and 77 in the testing set.

### 3.2 Screening for predictive factors

**Figure 1:**
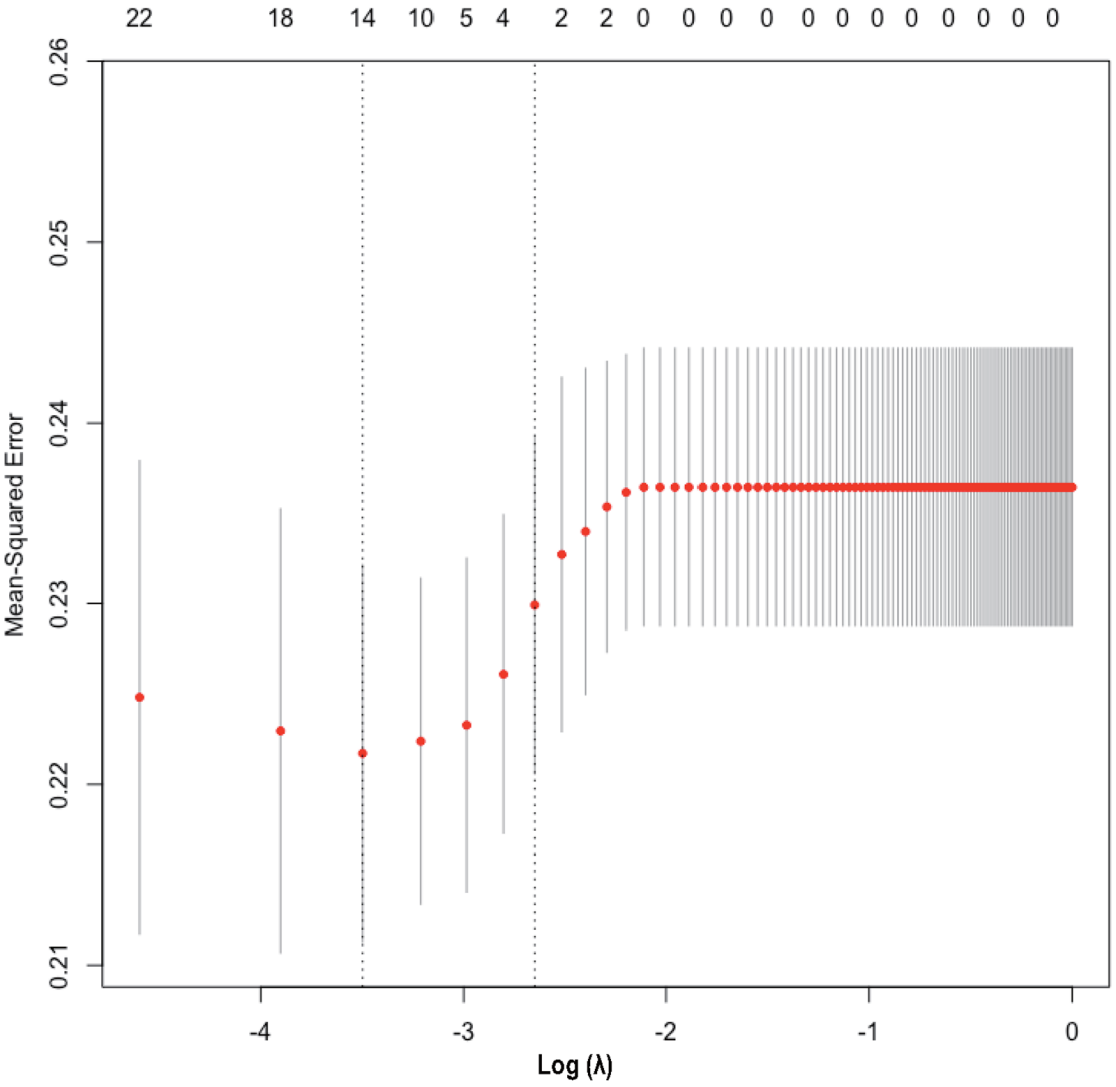
Cross-validation plot for Lasso regression model.

**Figure 2:**
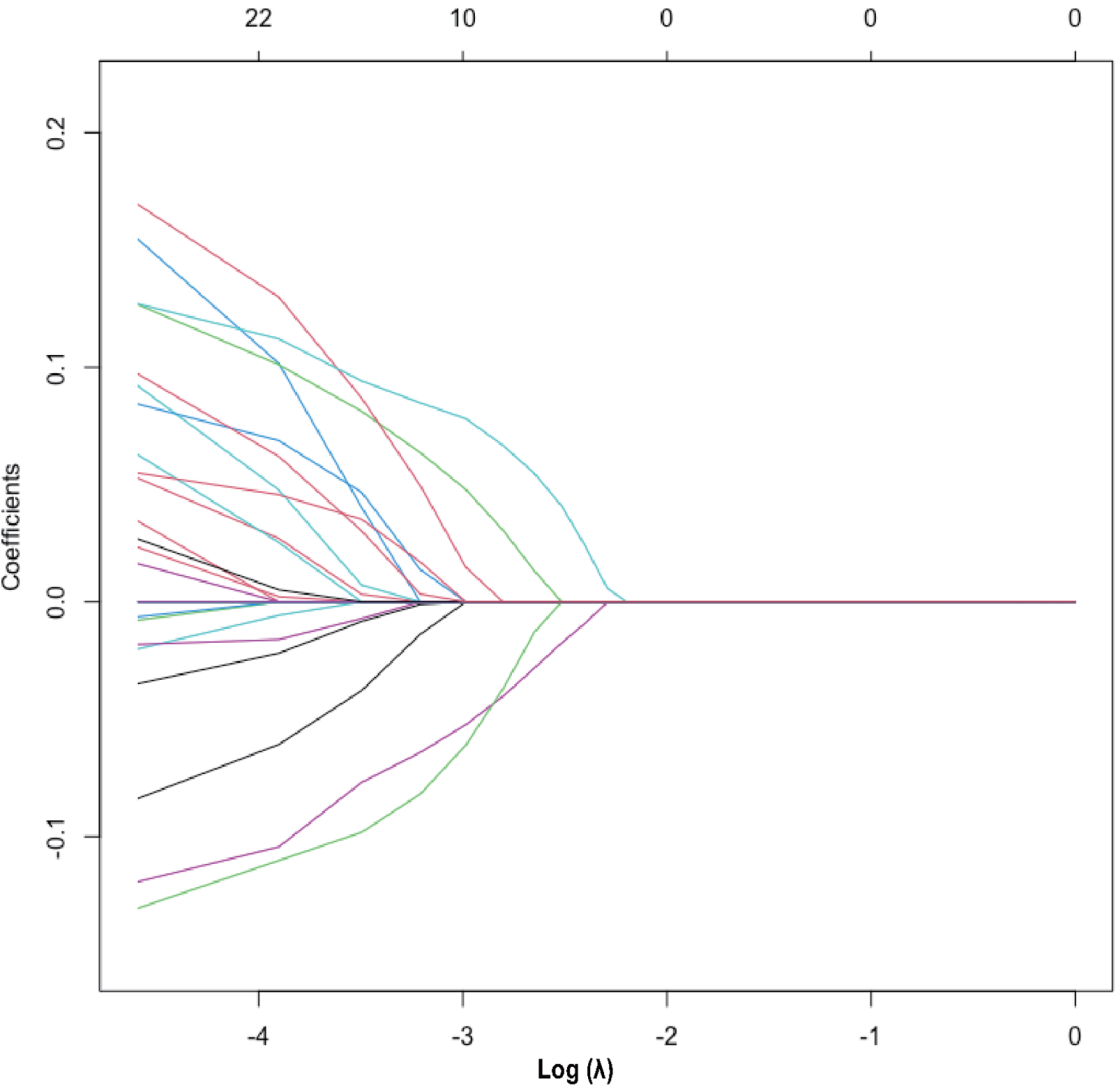
Lasso regression variable selection dynamic plot. Optimal parameter (lambda) selection in the Lasso model. The ordinate indicates the target parameter. The upper abscissa represents the number of non-zero coefficients, and the lower abscissa represents the log(*λ*).

The heatmap (Supplementary Figure S3) visually illustrates the correlations among multiple variables. Birth weight showed a positive correlation with gestational age (0.6813) and a negative correlation with total oxygen duration (−0.6261). Gestational age exhibited negative correlations with bronchopulmonary dysplasia (−0.5252), non-invasive ventilation duration (−0.6241), and total oxygen duration (−0.6944). Additionally, birth weight showed a positive correlation with invasive mechanical ventilation duration (0.5324). There was also a positive correlation between maternal education level and paternal education level (0.8005). Although these variables exhibited certain correlations, no significant collinearity was observed. Considering the findings from prior research and insights from clinical experts, as well as the importance of these variables, we did not exclude any variables, thereby ensuring a thorough analysis and understanding of all 33 variables included in the study. These findings were used to select variables for Lasso regression analysis (Figure 1), resulting in the selection of 14 significant predictive variables: neurodevelopmental delay, NEC surgery, gestational age, birth weight, 5-minute Apgar score, paternal education level, method of conception, premature rupture of membranes, chorioamnionitis, severe intraventricular hemorrhage, patent ductus arteriosus, cholestasis, hypothyroidism, and neonatal sepsis (Figure 2). LR analysis (Table 1) identified six independent factors that were significant at a 95% confidence level: gestational age (*P *= 0.0056), birth weight (*P *= 0.0015), premature rupture of membranes (*P *= 0.0069), patent ductus arteriosus (*P *= 0.0076), cholestasis (*P *= 0.0139), and neonatal sepsis (*P *= 0.0299). Comparison analysis revealed overlapping variables between the Lasso regression and LR analyses, indicating consistency between the two methods. To enhance the predictive power of the model, and based on clinical expert opinions, five additional variables were selected for subsequent prediction model construction: gender, intrauterine growth restriction, maternal age above 35 years, antenatal steroid use, and pregnancy-induced hypertension.

**Table 1:** Logistic regression (LR) model results for predicting EUGR.

Term	Estimate	Std.error	Statistic	*P*.value
(Intercept)	−2.6147	0.9420	−2.7756	0.0055
Sex	0.0149	0.3085	0.0484	0.9614
Neurodevelopmental delay	0.4500	0.3096	1.4534	0.1461
NEC	0.0046	0.5083	0.0090	0.9928
NEC surgery	0.9455	1.1052	0.8555	0.3923
PW^a^	0.7069	0.2550	2.7725	0.0056
BW^a^	−0.7346	0.2320	−3.1658	0.0015
5 min Apgar scores	−0.2427	0.1531	−1.5852	0.1129
IUGR	0.3610	0.4517	0.7992	0.4242
Mode of delivery	−0.1076	0.2997	−0.3588	0.7197
Maternal age over 35	−0.1980	0.3256	−0.6080	0.5432
Mother's education level	−0.2071	0.2426	−0.8537	0.3933
Father's education level	−0.1233	0.2395	−0.5147	0.6067
Mode of conception	−0.6041	0.3847	−1.5704	0.1163
PIH	0.2299	0.3412	0.6740	0.5003
PROM^a^	0.8458	0.3128	2.7039	0.0069
CAM	0.4976	0.5745	0.8662	0.3864
Antenatal steroids	0.4445	0.3794	1.1715	0.2414
NRDS	0.2862	0.3449	0.8297	0.4067
BPD	−0.1665	0.3667	−0.4539	0.6499
ICH over grade 3	1.1268	0.6177	1.8241	0.0681
PDA^a^	−0.8875	0.3324	−2.6697	0.0076
Hyperbilirubinemia	0.0037	0.4904	0.0076	0.9939
Cholestasis^a^	0.7993	0.3251	2.4586	0.0139
Anemia	0.0073	0.4797	0.0153	0.9878
Fungal infections	0.1818	1.2971	0.1402	0.8885
Hypoglycemia	0.3400	0.3751	0.9062	0.3648
Hypothyroidism	0.1344	1.3389	0.1004	0.9200
ROP	−0.0817	0.4316	−0.1893	0.8499
IMV	0.0258	0.2044	0.1260	0.8997
NMV	0.0455	0.2647	0.1719	0.8635
Total oxygenation time	0.2522	0.3231	0.7804	0.4351
Sepsis^a^	0.6980	0.3214	2.1716	0.0299

NEC, necrotizing enterocolitis; PW, pregnancy weeks; BW, birth weight; IUGR, Intrauterine Growth Restriction; PIH, Pregnancy-induced hypertension syndrome; PROM, premature rupture of membranes; CAM, chorioamnionitis; NRDS, neonatal respiratory distress syndrome; BPD, bronchopulmonary dysplasia; ICH, intracranial hemorrhage; PDA, patent ductus arteriosus; ROP, retinopathy of prematurity; IMV, invasive mechanical ventilation; NMV, noninvasive mechanical ventilation.

^a^ Indicates that the difference is statistically significant.

### 3.3 Development of risk prediction models

**Figure 3:**
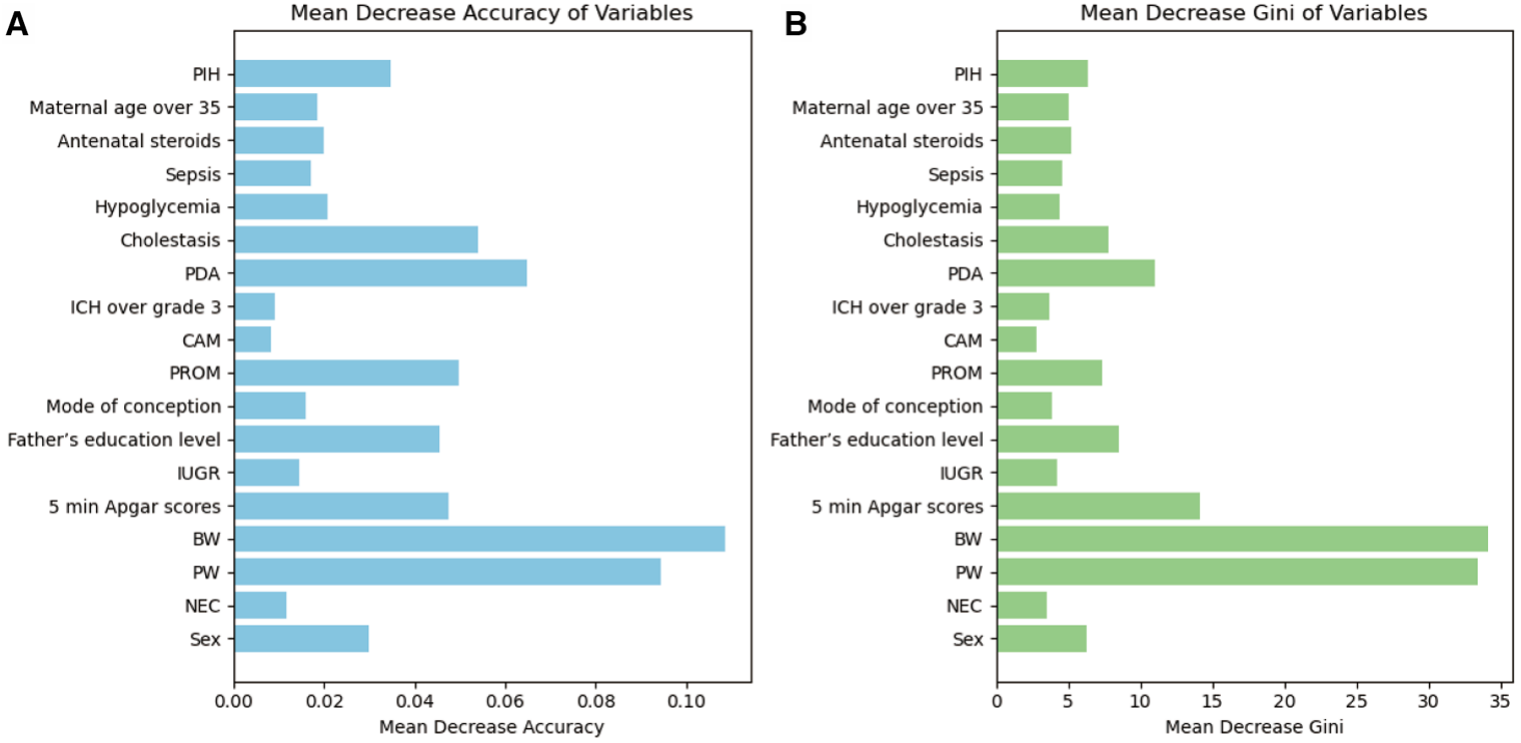
Importance of variables in Random Forest (RF) metrics. **(A)** Evaluation based on accuracy. **(B)** Evaluation based on Gini coefficient. PW, pregnancy weeks; BW, birth weight; PDA, patent ductus arteriosus; PROM, premature rupture of membranes; PIH, Pregnancy-induced hypertension syndrome; IUGR, Intrauterine Growth Restriction; ICH, intracranial hemorrhage; NEC, neonatal necrotizing enterocolitis; CAM, chorioamnionitis.

In the training set population, a RF model was constructed using the selected 19 variables as predictors, with the occurrence of EUGR as the outcome of interest. The RF model revealed that gestational age (0.0944), birth weight (0.1087), 5-min Apgar score (0.0476), patent ductus arteriosus (0.0648), and premature rupture of membranes (0.0499) were the top-ranked variables contributing significantly to the accuracy (Figure 3A). This observation was corroborated by the evaluation based on Gini coefficients (Figure 3B).

### 3.4 Comparison with other models and predictive accuracy

Alongside the RF model, a SVM model was constructed using the same variables, and a LR model was built using the six variables selected by logistic regression. Comparing the performance of the RF model, SVM model, and LR model, the RF model achieved the highest AUC of 0.62 (Supplementary Figure S4), indicating its superior predictive performance among the three models. Moreover, the RF model demonstrated the highest F1 score and accuracy, followed by the SVM model (Table 2). These findings suggest that machine learning methods have potential in constructing clinical risk prediction models and provide evidence for the suitability of the RF model in predicting EUGR (Table 2).

**Table 2:** AUC, F1 score, and Accuracy of three predictive models on the test set.

Model	AUC	F1 score	Accuracy
Random forest	0.6224	0.7963	0.7272
Multivariate logistic regression model	0.5466	0.7434	0.6234
Support vector machine	0.5000	0.7778	0.6364

**Figure 4:**
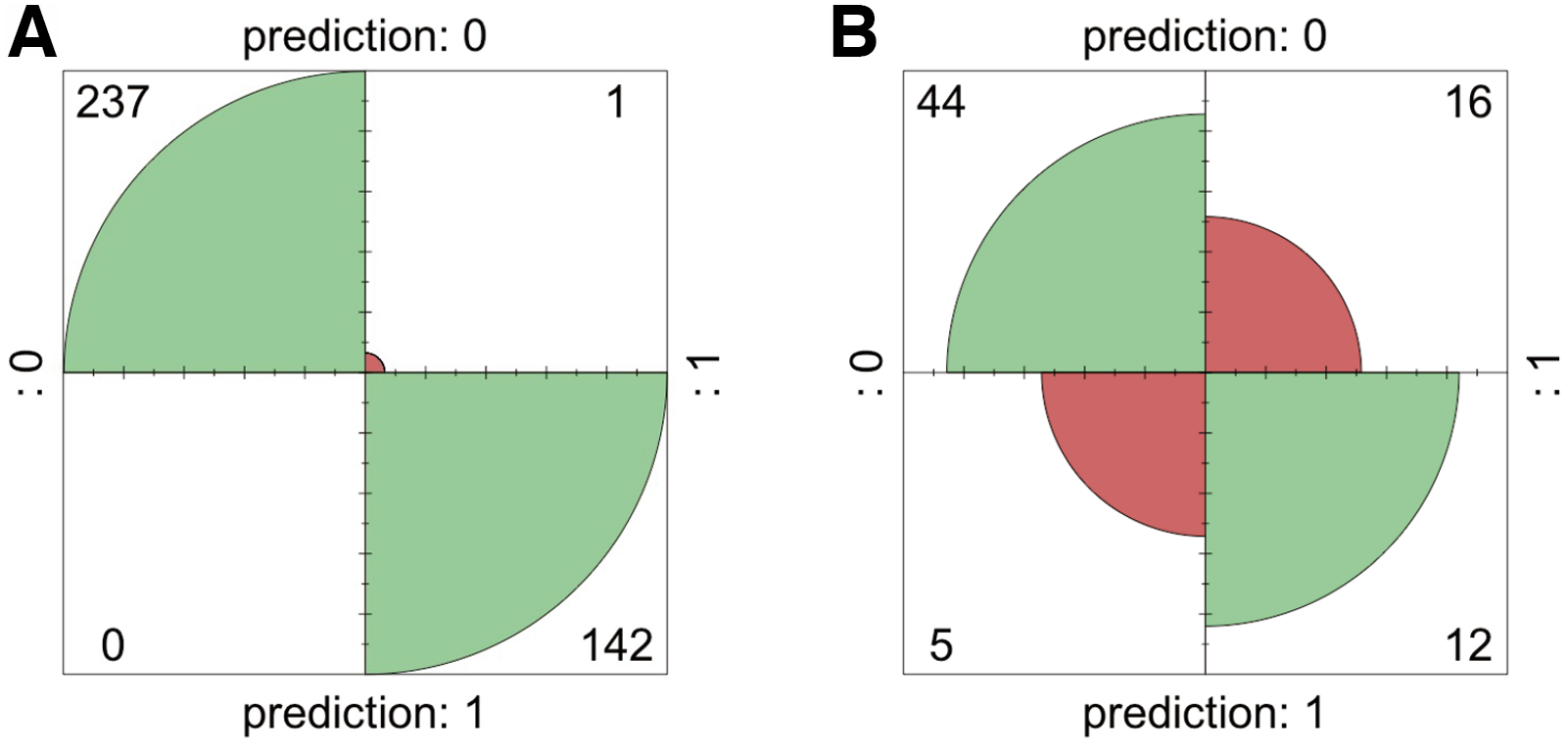
Confusion matrix for the training and testing sets. **(A)** Training set. **(B)** Testing set.

Internal validation using bootstrap resampling (380 iterations) demonstrated the stability of the predictive model. Confusion matrices were generated to assess the performance of the RF model, comparing the predicted classifications with the true classifications in the training set (Figure 4A). The RF model achieved an accuracy of 99.99% in the training set and 72.72% in the testing set, validating the model's reliability externally (Figure 4B).

**Figure 5:**
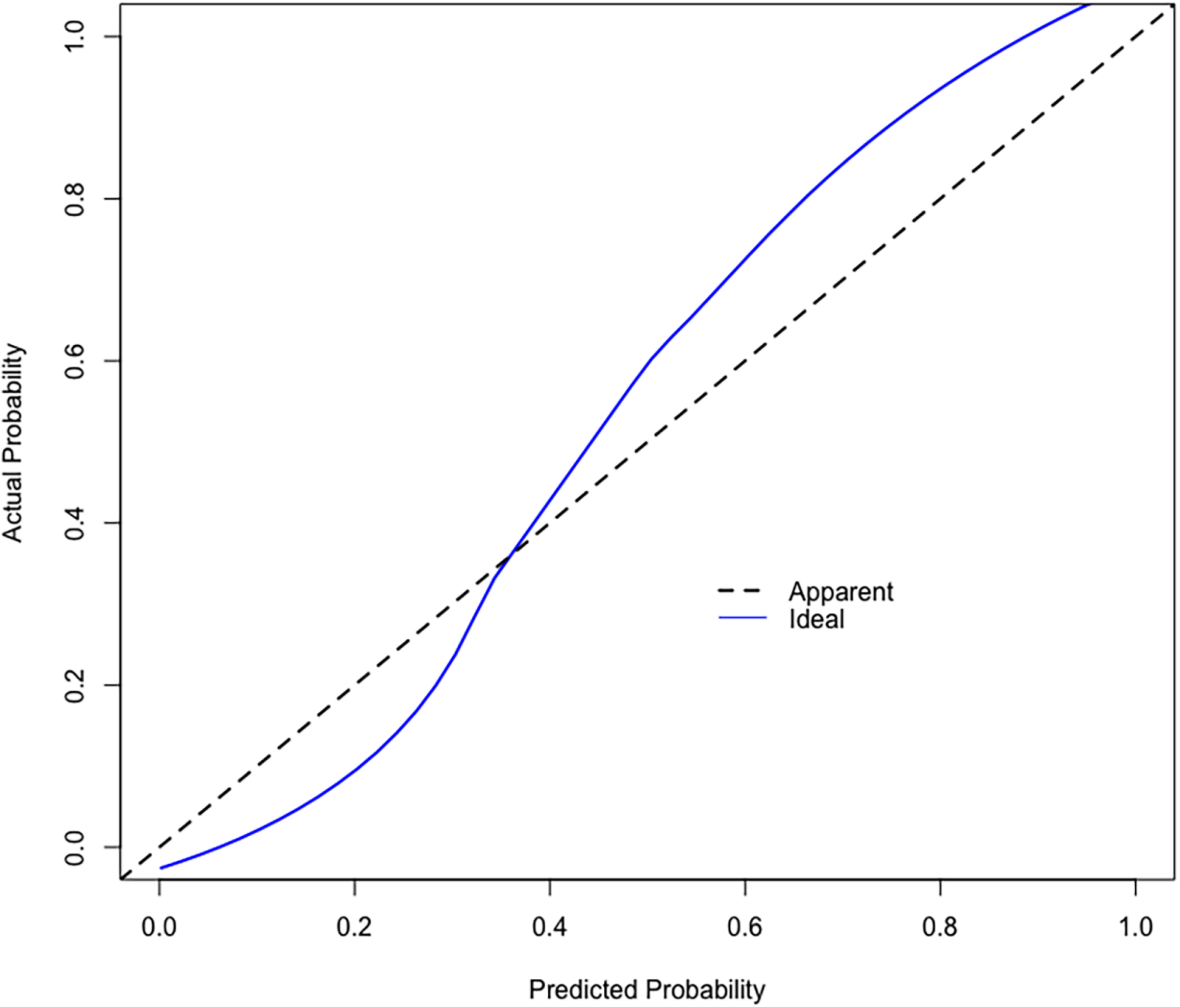
Calibration curve of the Random Forest (RF) model.

**Figure 6:**
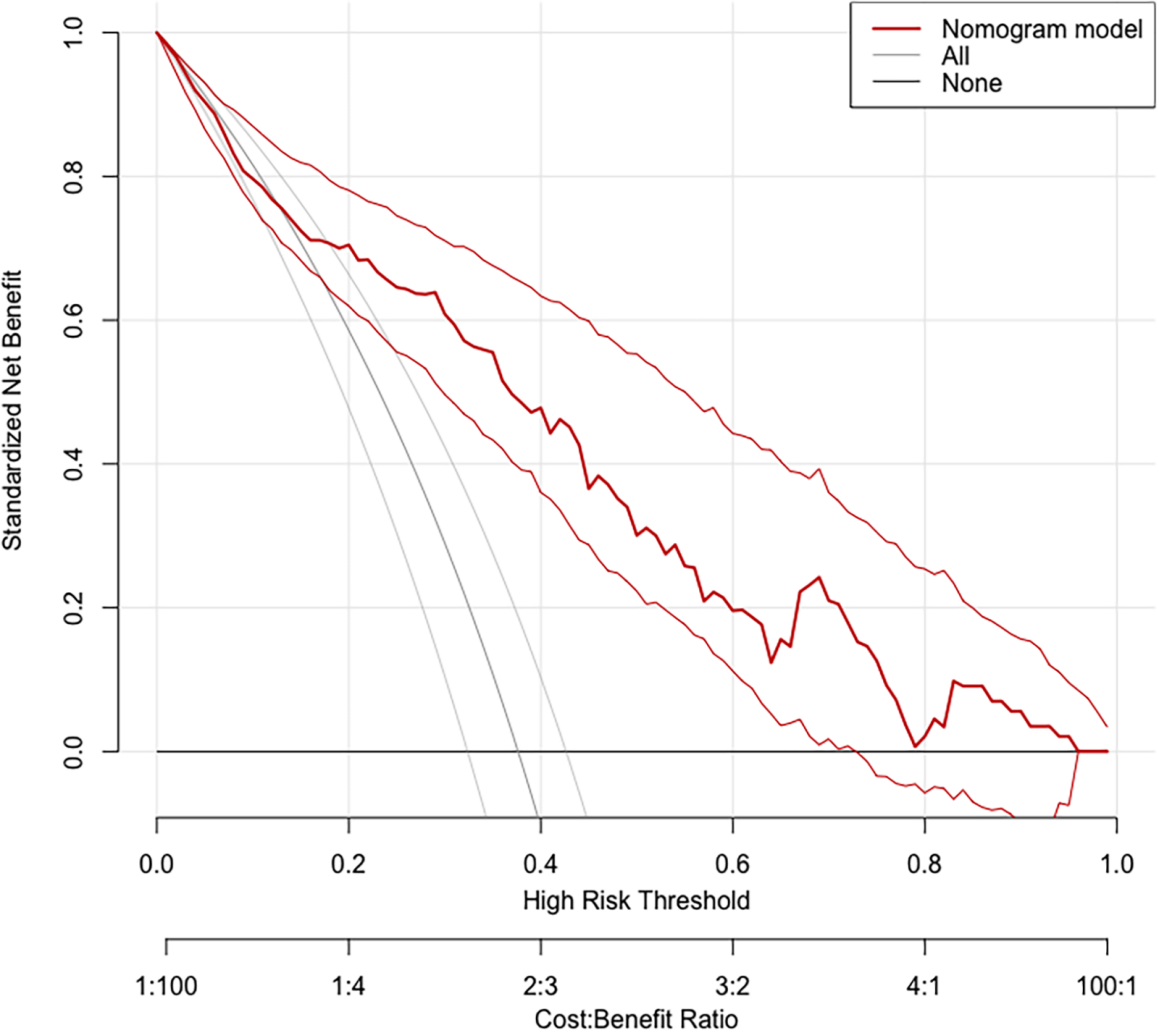
Decision Curve Analysis (DCA) demonstrating the clinical applicability of the Random Forest (RF) model.

Based on the calibration curve plotted using the training set, when the risk of EUGR is low (<0.4), the model predicts probabilities slightly lower than the actual probabilities (Figure 5). Conversely, when the risk of EUGR is high (≥0.4), the model's predictions are more conservative than the actual probabilities, indicating cautious predictions by the model (Figure 5). The overall calibration curve closely approximates the ideal curve, fluctuating near the 45-degree diagonal, suggesting good overall performance of the constructed random forest model. Using the DCA curve to evaluate the clinical applicability of the predictive model across the entire population, the DCA curve lies above the diagonal and horizontal lines, indicating that utilizing the model developed in this study to predict EUGR in premature infants born before 34 weeks gestation would yield greater net benefit (Figure 6).

## 4 Discussion

In this study, clinically collinear variables in the training set were excluded based on the Pearson correlation coefficient. Lasso regression and LR analyses were performed to determine the final predictive factors. A RF prediction model was established using a total of 19 predictive factors, including gestational age and birth weight, achieving an accuracy of 99.99%, sensitivity of 83.33%, and specificity of 53.85%. Internal validation was conducted using bootstrap resampling, and the model demonstrated good stability in the test set queue during external validation, showing higher reliability compared to SVM and LR models. Calibration curves were plotted to assess the probability prediction performance of the forecasting model, and DCA was conducted to validate the clinical efficacy of the random forest model.

The study analyzed the correlation between variables. It was observed that birth weight had a positive correlation with gestational age and the duration of invasive mechanical ventilation, while the total oxygen duration had a negative correlation with birth weight. Although correlation does not imply causation, and an association between two variables does not necessarily indicate direct influence, examining correlations between variables aids in exploring predictive outcomes, identifying risk factors, and providing valuable information for clinical decision-making.

Both LR and Lasso regression analyses identified that gestational age, birth weight, premature rupture of membranes, patent ductus arteriosus, cholestasis, and neonatal sepsis were the main predictive variables for EUGR. Previous studies have highlighted the importance of gestational age and birth weight as predictive factors for EUGR. Experts have emphasized that infants with very low birth weight, those born small for their gestational age, and those born extremely preterm are at a higher risk of developing EUGR (33). Research by Zhang (11) also clearly demonstrated that low birth weight and inadequate gestational age were key risk factors for EUGR. The delayed postnatal physiological weight loss, combined with poor nutrition intake and complications, exacerbates the delayed growth. Therefore, providing appropriate feeding and ensuring sufficient calorie intake to premature infants as early as possible, striving to shorten the duration and magnitude of physiological weight loss, may effectively reduce the occurrence of EUGR.

The RF prediction model indicated that gestational age, birth weight, 5-minute Apgar score, patent ductus arteriosus, and premature rupture of membranes were important variables in determining the classification. Experts such as Menon have pointed out that premature rupture of membranes (34) is associated with the occurrence of severe complications in the short- and long-term, including neonatal mortality, periventricular leukomalacia, bronchopulmonary dysplasia, necrotizing enterocolitis (NEC), retinopathy of prematurity, and adverse neurological outcomes (35, 36). The present study also identified premature rupture of membranes as one of the most important predictive factors for EUGR, consistent with previous literature. Premature rupture of membranes can lead to infections, abnormal amniotic fluid, and cervical insufficiency, all of which are linked to EUGR (37). Therefore, effectively preventing and treating premature rupture of membranes may be an important measure for preventing EUGR and improving short-term growth and long-term developmental outcomes in preterm infants.

Additionally, this study also demonstrated an association between cholestasis, neonatal sepsis (38), patent ductus arteriosus (39), and EUGR. Due to the immaturity of the digestive system, preterm infants may experience delayed establishment of intestinal microbiota (40). This makes preterm infants more prone to feeding intolerance, cholestasis, NEC, neonatal sepsis, adverse neurological development, and allergic diseases, which can affect nutrient absorption (41) and contribute to EUGR. Key aspects in the treatment of sepsis or cholestasis include focusing on nutritional support, monitoring growth, early stimulation, managing patent ductus arteriosus, maintaining intestinal health, controlling infections, and facilitating multidisciplinary teamwork to ensure optimal treatment and rehabilitation for patients, thereby avoiding the occurrence of EUGR. It is important to emphasize that in the clinical treatment of EUGR patients, a comprehensive approach that integrates established EUGR-related factors from current research and customizes treatment and nutritional support based on the individual growth status of each patient can effectively enhance growth and development in EUGR patients. Breastfeeding is recommended in clinical practice, and early initiation of enteral nutrition is advocated to rapidly achieve full enteral feeding, effectively increases cumulative calorie intake during the first week, reduces the occurrence of EUGR and complications such as cholestasis, while avoiding the risks of other complications in preterm infants (42).

This study has certain limitations: The data used to establish the disease prediction model were sourced from a single center, lacking validation from external hospital cohorts; The sample size was relatively modest. Therefore, it is necessary to include external hospital cohorts for validation and increase the sample size in future studies. Further analysis with follow-up results can also enhance the predictive performance, clinical applicability, and generalizability of the model.

In summary, this study developed and validated a risk prediction model for EUGR in preterm infants born before 34 weeks of gestation. The predictive model provides clinicians with a scientifically effective tool for preventing and early intervention of EUGR in this population, and it has a significant reference value for accurate diagnosis, treatment, and objective assessment of EUGR patients.

## Data Availability

The datasets presented in this article are not readily available because due to concerns regarding patient privacy, the handling of clinical data obtained from our healthcare institution necessitates careful consideration. Requests to access the datasets should be directed to YL, angelliyan2008@yeah.net&lt.

## References

[1] Vogel JP Chawanpaiboon S Moller A-B Watananirun K Bonet M Lumbiganon P. The global epidemiology of preterm birth. Best Pract Res Clin Obstet Gynaecol. (2018) 52:3–12. 10.1016/j.bpobgyn.2018.04.003 29779863

[2] Liu L Oza S Hogan D Chu Y Perin J Zhu J Global, regional, and national causes of under-5 mortality in 2000–15: an updated systematic analysis with implications for the sustainable development goals. Lancet. (2016) 388(10063):3027–35. 10.1016/S0140-6736(16)31593-8 27839855 PMC5161777

[3] Rundell K Panchal B. Preterm labor: prevention and management. Am Fam Physician. (2017) 95(6):366–72. 28318214

[4] Sakurai M Itabashi K Sato Y Hibino S Mizuno K. Extrauterine growth restriction in preterm infants of gestational age ≤ 32 weeks. Pediatr Int. (2008) 50(1):70–5. 10.1111/j.1442-200X.2007.02530.x 18279209

[5] Shen W Wu F Mao J Liu L Chang Y-M Zhang R Analysis of ‘true extrauterine growth retardation’ and related factors in very preterm infants—a multicenter prospective study in China. Front Pediatr. (2022) 10:876310. 10.3389/fped.2022.876310 36210927 PMC9534122

[6] Clark RH Thomas P Peabody J. Extrauterine growth restriction remains a serious problem in prematurely born neonates. Pediatrics. (2003) 111(5):986–90. 10.1542/peds.111.5.986 12728076

[7] Khasawneh W Khassawneh M Mazin M Al-Theiabat M Alquraan T. Clinical and nutritional determinants of extrauterine growth restriction among very low birth weight infants. Int J Gen Med. (2020) 13:1193–200. 10.2147/IJGM.S284943 33239903 PMC7682780

[8] Ordóñez-Díaz MD Pérez-Navero JL Flores-Rojas K Olza-Meneses J Muñoz-Villanueva MC Aguilera-García CM Prematurity with extrauterine growth restriction increases the risk of higher levels of glucose, low-grade of inflammation and hypertension in prepubertal children. Front Pediatr. (2020) 8:180. 10.3389/fped.2020.00180 32373566 PMC7186313

[9] Martínez-Jiménez M Gómez-García F Gil-Campos M Pérez-Navero J. Comorbidities in childhood associated with extrauterine growth restriction in preterm infants: a scoping review. Eur J Pediatr. (2020) 179:1255–65. 10.1007/s00431-020-03613-8 32096070

[10] Pampanini V Boiani A De Marchis C Giacomozzi C Navas R Agostino R Preterm infants with severe extrauterine growth retardation (EUGR) are at high risk of growth impairment during childhood. Eur J Pediatr. (2015) 174:33–41. 10.1007/s00431-014-2361-z 24953378

[11] Zhao T Feng H-M Caicike B Zhu Y-P. Investigation into the current situation and analysis of the factors influencing extrauterine growth retardation in preterm infants. Front Pediatr. (2021) 9:643387. 10.3389/fped.2021.643387 33996689 PMC8119632

[12] Gidi NW Goldenberg RL Nigussie AK McClure E Mekasha A Worku B Incidence and associated factors of extrauterine growth restriction (EUGR) in preterm infants, a cross-sectional study in selected NICUs in Ethiopia. BMJ Paediatr Open. (2020) 4(1):e000765. 10.1136/bmjpo-2020-000765 33094173 PMC7552851

[13] Coverston CR Schwartz R. Extrauterine growth restriction: a continuing problem in the NICU. MCN Am J Matern Child Nurs. (2005) 30(2):101–6. 10.1097/00005721-200503000-00006 15775804

[14] Radmacher PG Looney SW Rafail ST Adamkin DH. Prediction of extrauterine growth retardation (EUGR) in VVLBW infants. J Perinatol. (2003) 23(5):392–5. 10.1038/sj.jp.7210947 12847535

[15] Tripepi G Heinze G Jager KJ Stel VS Dekker FW Zoccali C. Risk prediction models. Nephrol Dial Transplant. (2013) 28(8):1975–80. 10.1093/ndt/gft095 23658248

[16] Black JE Kueper JK Williamson TS. An introduction to machine learning for classification and prediction. Fam Pract. (2023) 40(1):200–4. 10.1093/fampra/cmac104 36181463

[17] Peterson ED. Machine learning, predictive analytics, and clinical practice: can the past inform the present? JAMA. (2019) 322(23):2283–4. 10.1001/jama.2019.17831 31755902

[18] Ij H. Statistics versus machine learning. Nat Methods. (2018) 15(4):233. 10.1038/nmeth.4642 30100822 PMC6082636

[19] Al-Hindawi A Abdulaal A Rawson TM Alqahtani SA Mughal N Moore LS. COVID-19 prognostic models: a pro-con debate for machine learning vs. traditional statistics. Front Digit Health. (2021) 3:637944. 10.3389/fdgth.2021.637944 35005694 PMC8734592

[20] Shao X Ye H Qiu X. Practice of neonatology. Version. (2011) 4:699–06.

[21] Subspecialty Group of Neonatology SG. Expert consensus on the diagnosis and management of neonatal sepsis (version 2019). Zhonghua Er Ke Za Zhi. (2019) 57(4):252–7. 10.3760/cma.j.issn.0578-1310.2019.04.005 30934196

[22] Shane AL Sánchez PJ Stoll BJ. Neonatal sepsis. Lancet. (2017) 390(10104):1770–80. 10.1016/S0140-6736(17)31002-4 28434651

[23] Sweet DG Carnielli VP Greisen G Hallman M Klebermass-Schrehof K Ozek E European consensus guidelines on the management of respiratory distress syndrome: 2022 update. Neonatology. (2023) 120(1):3–23. 10.1159/000528914 36863329 PMC10064400

[24] Chiang MF Quinn GE Fielder AR Ostmo SR Chan RP Berrocal A International classification of retinopathy of prematurity. Ophthalmology. (2021) 128(10):e51–68. 10.1016/j.ophtha.2021.05.031 34247850 PMC10979521

[25] Walsh MC Kliegman RM. Necrotizing enterocolitis: treatment based on staging criteria. Pediatr Clin N Am. (1986) 33(1):179–201. 10.1016/S0031-3955(16)34975-6 PMC7131118 3081865

[26] Papile L-A Burstein J Burstein R Koffler H. Incidence and evolution of subependymal and intraventricular hemorrhage: a study of infants with birth weights less than 1,500 gm. J Pediatr. (1978) 92(4):529–34. 10.1016/S0022-3476(78)80282-0 305471

[27] Sweet DG Carnielli V Greisen G Hallman M Ozek E Te Pas A European consensus guidelines on the management of respiratory distress syndrome–2019 update. Neonatology. (2019) 115(4):432–50. 10.1159/000499361 30974433 PMC6604659

[28] Impey L Child T. Obstetrics and Gynaecology. New York: John Wiley & Sons (2017).

[29] Jianping D. Early diagnosis of intrauterine infection. Chin J Pract Gynecol Obstet. (2014) 30:418–21.

[30] Huang J Ling CX. Using AUC and accuracy in evaluating learning algorithms. IEEE Trans Knowl Data Eng. (2005) 17(3):299–310. 10.1109/TKDE.2005.50

[31] Moosavi SM Ghassabian S. Linearity of calibration curves for analytical methods: a review of criteria for assessment of method reliability. Calibration and Validation of Analytical Methods—a Sampling of Current Approaches. (2018). p. 109–27. 10.5772/intechopen.72932

[32] Vickers AJ Elkin EB. Decision curve analysis: a novel method for evaluating prediction models. Med Decis Making. (2006) 26(6):565–74. 10.1177/0272989x06295361 17099194 PMC2577036

[33] Makker K Ji Y Hong X Wang X. Antenatal and neonatal factors contributing to extra uterine growth failure (EUGR) among preterm infants in Boston Birth Cohort (BBC). J Perinatol. (2021) 41(5):1025–32. 10.1038/s41372-021-00948-4 33589730 PMC7883994

[34] Menon R Richardson LS. Preterm prelabor rupture of the membranes: a disease of the fetal membranes. Semin Perinatol. (2017) 41(7):409–19. 10.1053/j.semperi.2017.07.012 28807394 PMC5659934

[35] Zhang G Yang M Wu Z Lam W Lian C Zhao G Changes in the incidence of retinopathy of prematurity in extremely low birth weight infants in south China from 2004 to 2018. Ophthalmic Epidemiol. (2021) 28(4):359–64. 10.1080/09286586.2020.1826542 33021141

[36] Woo SJ Park JY Hong S Kim YM Park YH Lee YE Inflammatory and angiogenic mediators in amniotic fluid are associated with the development of retinopathy of prematurity in preterm infants. Invest Ophthalmol Visual Sci. (2020) 61(5):42. 10.1167/iovs.61.5.42 PMC7405804 32446247

[37] Zhang Z Mei L Li L Xiao J Wu X Yuan Y. Maternal and neonatal outcomes of twin pregnancies complicated by gestational diabetes mellitus. Endocrine. (2023) 84(2):388–98. 10.1007/s12020-023-03588-0 37946069 PMC11076322

[38] Yancey MK Duff P Kubilis P Clark P Frentzen BH. Risk factors for neonatal sepsis. Obstet Gynecol. (1996) 87(2):188–94. 10.1016/0029-7844(95)00402-5 8559521

[39] Velazquez DM Reidy KJ Sharma M Kim M Vega M Havranek T. The effect of hemodynamically significant patent ductus arteriosus on acute kidney injury and systemic hypertension in extremely low gestational age newborns. J Matern Fetal Neonatal Med. (2019) 32(19):3209–14. 10.1080/14767058.2018.1460349 29642731

[40] Lee JK-F Hern Tan LT Ramadas A Ab Mutalib N-S Lee L-H. Exploring the role of gut bacteria in health and disease in preterm neonates. Int J Environ Res Public Health. (2020) 17(19):6963. 10.3390/ijerph17196963 32977611 PMC7579082

[41] Hu F Tang Q Wang Y Wu J Ruan H Lu L Analysis of nutrition support in very low-birth-weight infants with extrauterine growth restriction. Nutr Clin Pract. (2019) 34(3):436–43. 10.1002/ncp.10210 30421458 PMC7379204

[42] Gao L Shen W Wu F Mao J Liu L Chang Y-M Effect of early initiation of enteral nutrition on short-term clinical outcomes of very premature infants: a national multicenter cohort study in China. Nutrition. (2023) 107:111912. 10.1016/j.nut.2022.111912 36577163

