# Supplementary material for: Construction and validation of a risk prediction model for extrauterine growth restriction in preterm infants born at gestational age less than 34 weeks
Source: Front Pediatr. 2024 Sep 18;12:1381193. doi: 10.3389/fped.2024.1381193 (PMC11445175; doi:10.3389/fped.2024.1381193)

Supplementary Material for "Construction and Validation of a Risk Prediction Model for Extrauterine Growth Restriction in Preterm Infants Born at Gestational Age Less Than 34 Weeks"

Fig S1. Inclusion and Exclusion Criteria Flowchart.

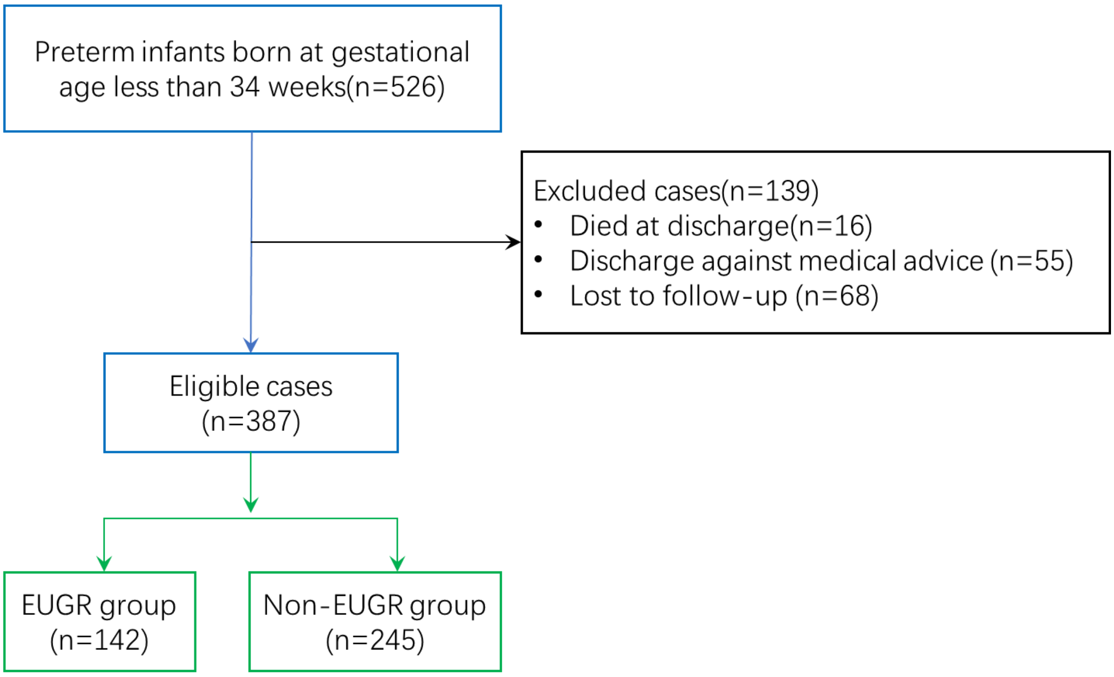

Fig S2. Flowchart for Constructing a Random Forest Prediction Model.

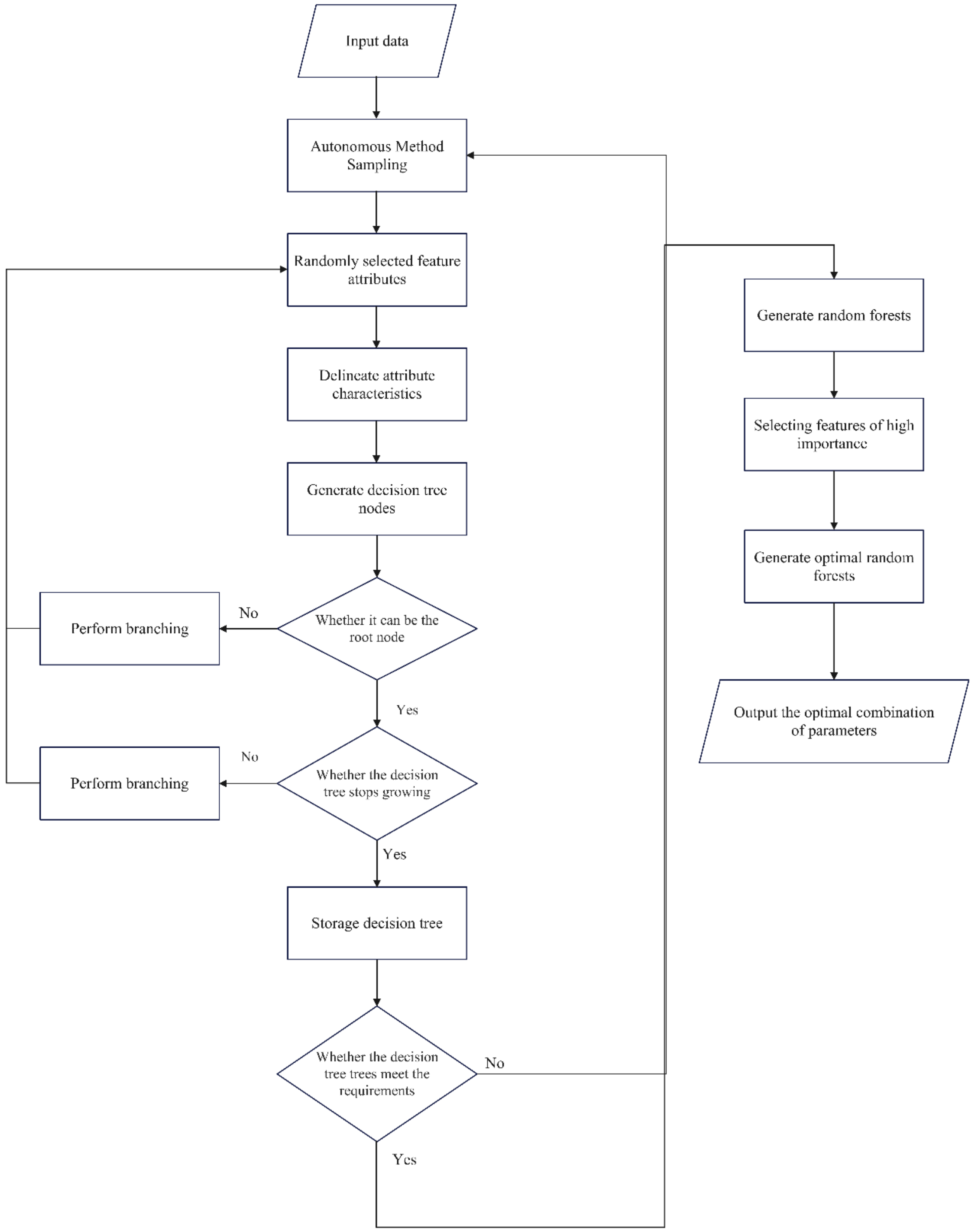

Fig S3. The Heatmap for Variable Correlation.

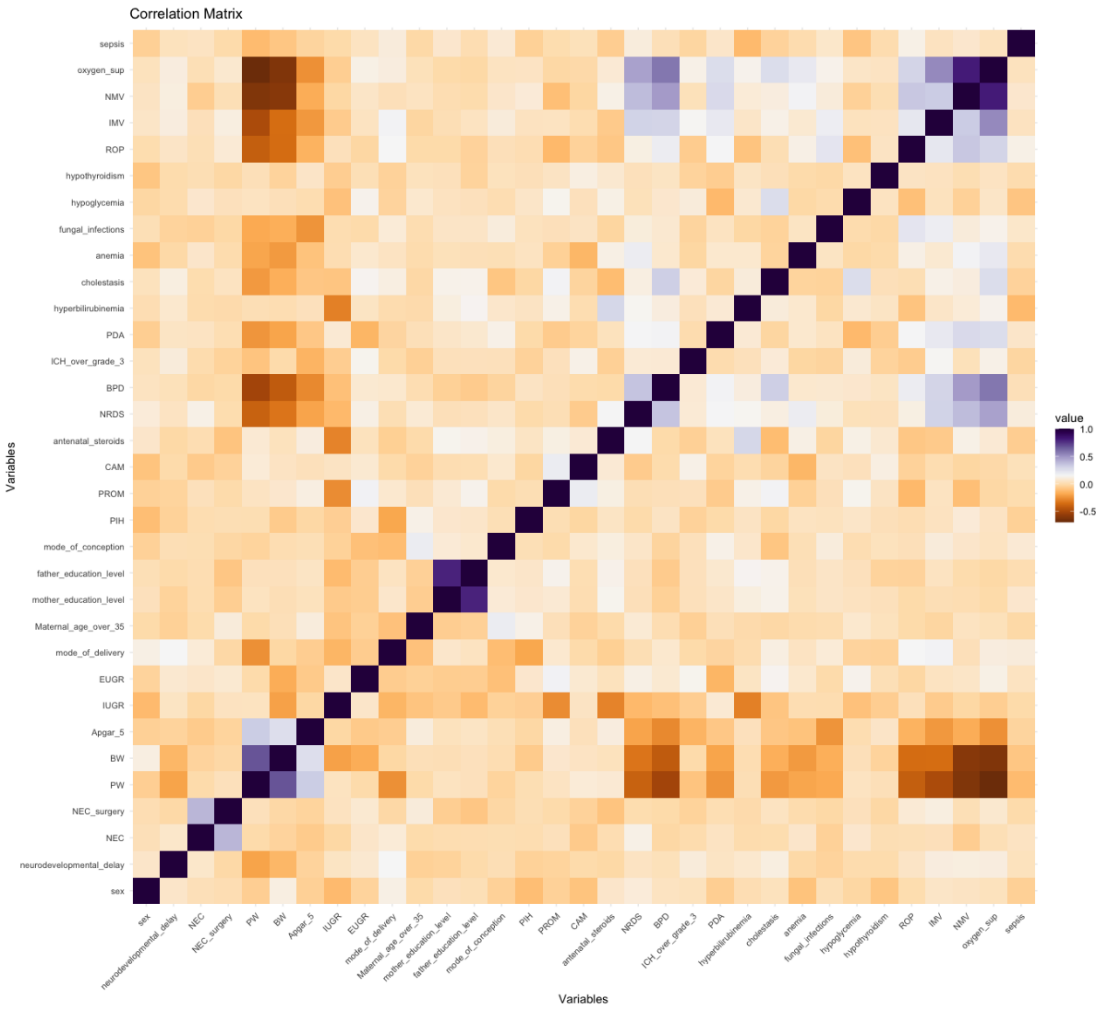

Fig S4. The AUC curves for Three Predictive Models.

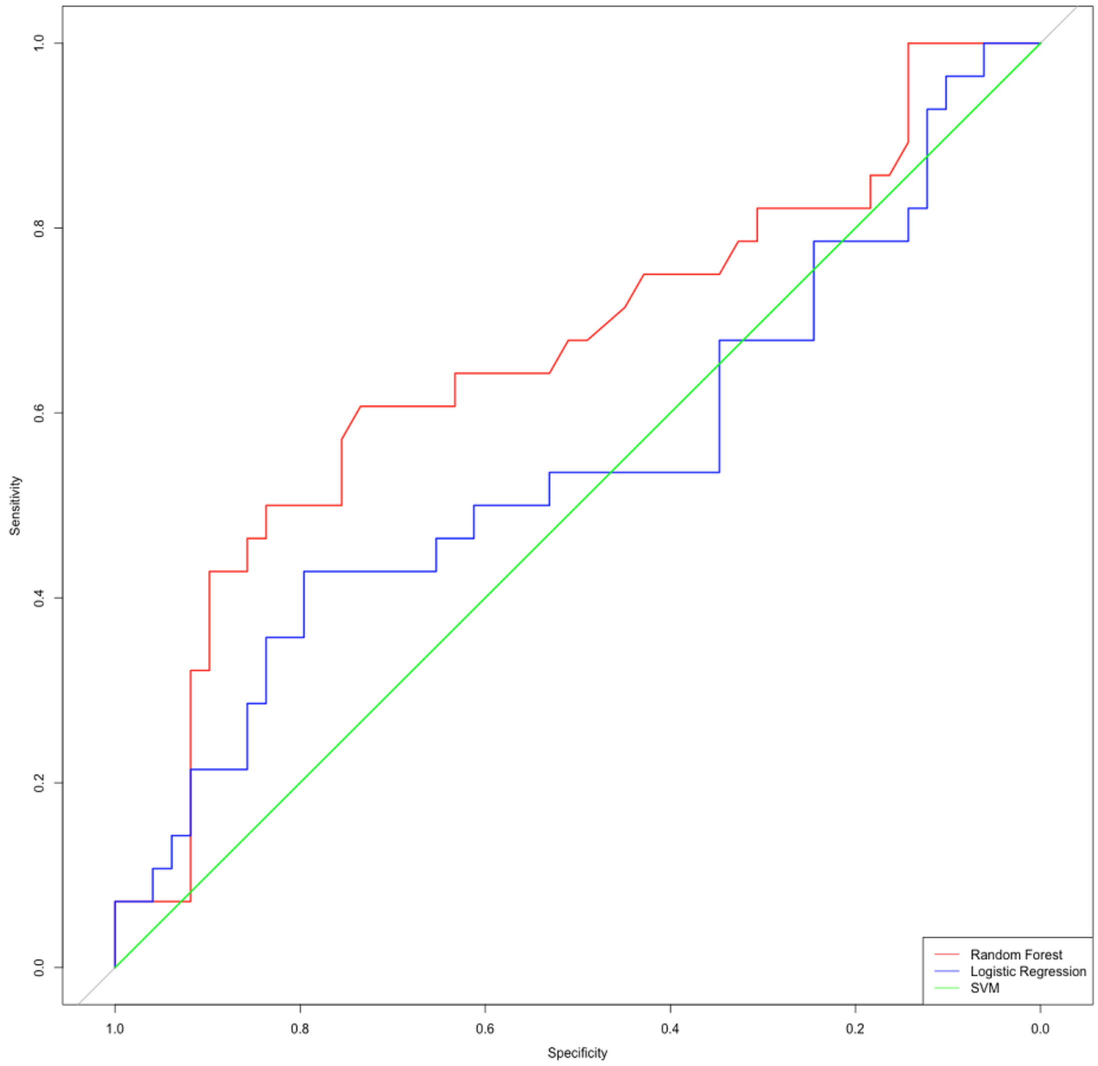

Supplement: Supplementary file 1 [file Datasheet1.pdf]
